# On the Ejection of Filaments of Polymer Solutions Triggered by a Micrometer-Scale Mixing Mechanism

**DOI:** 10.3390/ma14123399

**Published:** 2021-06-19

**Authors:** Fernando Marín-Brenes, Jesús Olmedo-Pradas, Alfonso M. Gañán-Calvo, Luis Modesto-López

**Affiliations:** Department of Aerospace Engineering and Fluid Mechanics, University of Seville, Camino de los Descubrimientos s/n, 41092 Seville, Spain; fernandomarin.fmb@gmail.com (F.M.-B.); jesusolmedopradas@gmail.com (J.O.-P.); amgc@us.es (A.M.G.-C.)

**Keywords:** flow blurring, poly(ethylene oxide), filaments, scaling law, viscosity, turbulence, Kolmogorov’s theory, boundary layer thickness, liquid flow rate

## Abstract

Polymer filaments constitute precursor materials of so-called fiber mats, ubiquitous structures across cutting-edge technological fields. Thus, approaches that contribute to large-scale production of fibers are desired from an industrial perspective. Here, we use a robust liquid atomization device operated at relatively high flow rates, ~20 mL/min, as facilitating technology for production of multiple polymer filaments. The method relies on a turbulent, energetically efficient micro-mixing mechanism taking place in the interior of the device. The micro-mixing is triggered by radial implosion of a gas current into a liquid feeding tube, thus resulting in breakup of the liquid surface. We used poly(ethylene oxide) solutions of varying concentrations as test liquids to study their fragmentation and ejection dynamics employing ultra-high speed imaging equipment. Taking an energy cascade approach, a scaling law for filament diameter was proposed based on gas pressure, liquid flow rate and viscosity. We find that a filament dimensionless diameter, $D_{f}^{*}$, scales as a non-dimensional liquid flow rate $Q^{*}$ to the 1/5. The study aims to elucidate the underlying physics of liquid ejection for further applications in material production.

## 1. Introduction

The role of polymers in flow dynamics has been the subject of intense research for a few decades since the discovery of the so-called drag reduction phenomenon. Recent review articles and the rise of scientific reports dealing with such topic evidence its current relevance [1,2]. The plethora of fluidic phenomena stemming from the chemical composition, microstructure, and fabrication methods of polymers are active fields of research. Furthermore, these macromolecules are ubiquitous in synthesis processes both in traditional industrial fields and in contemporary approaches for production of materials with tailored dimensions and engineered properties. One of the common application of polymers is as raw components in the synthesis of micro- and nanofibers and their derivatives, so-called nonwoven mats [3,4,5]. Perhaps, the most representative example of a method for fiber fabrication is the electrospinning, in which a jet of a polymeric solution is pulled out from a bulk by electric stress acting on its surface, and is simultaneously elongated in-flight as a result of a high electric potential [6,7,8,9,10]. In the solution, the polymer concentration is a critical parameter that strongly influences the liquid’s rheology due to stearic conformation and interaction of the polymer chains, thus resulting in formation of coils. Other methods for processing of polymer solutions include melt blow, flow focusing, and liquid atomization strategies [11,12,13].

**Figure 1:**
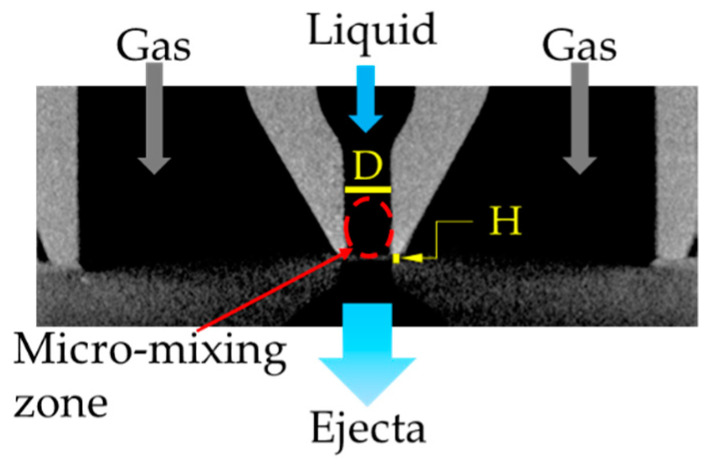
Cross-sectional view of a typical micro-mixing device. The gas implodes radially into the tube that supplies the liquid, thus generating a micro-mixing zone. D = 700 μm and H = 100 μm.

Here, we present an energetically efficient approach for ejection of liquid structures based on a mixing mechanism at the micrometer length scale. The micro-mixing is the fundamental feature of so-called Flow Blurring (FB) technology, which is a liquid atomization approach. The FB atomizers consist of concentric tubes where the liquid and gas are fed through the inner and external conduits, respectively, both fluids coinciding at the tip of the liquid feeding tube [14]. Subsequently, the gas stream radially implodes into the inner tube, thus triggering a bubbly turbulent motion in the region near its tip (see for instance Figure 3 in ref. [14] or Figure 1 in ref. [15]). Eventually a quasi-steady state is reached in which the gas motion is balanced by the inertia of the liquid, thus creating a gas cavity that, based on observations, is maintained due to a continuous supply of both phases [16,17,18]. Such a bubbling motion can fragment the liquid bulk, resulting in smaller ejecta, typically in the form of droplets [14,17]. Gañán-Calvo determined that there exists a fundamental geometrical parameter Ψ=H/D<0.25 that establishes the occurrence of the micro-mixing phenomenon in these type of atomizers [14]. H is the distance from the tip of the inner tube (liquid feeding tube) to the discharge orifice and D is the diameter of that orifice, which is typically the diameter of the inner tube too (see Figure 1). Flow Blurring atomizers have generally been used to produce droplets and aerosols from Newtonian liquids such as water, alcohols, polyols, glycols, oils or fuels in general [14,17,19,20,21,22]. Nevertheless, we have extended its application to the fragmentation of polymeric solutions, which exhibit a more complex rheology.

**Figure 2:**
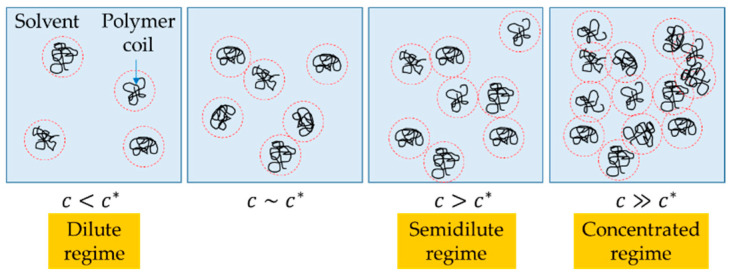
Representation of polymer solutions based on concentration. $c^{*}$ is the coil overlap concentration.

The rheology of polymer solutions depends on several factors such as polymer molecular weight, concentration, type of solvent, temperature, and pressure. Generally, based on concentration polymer solutions are classified as: dilute, semidilute, and concentrated [22], as depicted in the illustration of Figure 2. In the first case, in dilute solutions, short-range interactions of polymer chains cause them to assemble into coils, which are intricate structures that look like “spaghetti”. These coils do not interact significantly with each other because in a dilute regime the solution concentration, c, is smaller than a critical concentration above which the coils overlap, called the coil overlap concentration, and denoted by $c^{*}$. In the semidilute regime, however, the polymer coils start to interact because $c∼c^{*}$, and thus they experience overlapping. In this regime, depending on the interactions of the coils, the solutions may also be divided into semidilute unentangled and semidilute entangled solutions. The entanglements develop from the interpenetration of random-coil polymer chains and may be quantified by an entanglement concentration, $c_{e}$. Finally, in the case of concentrated solutions, $c\gg c^{*}$ and polymer coils strongly interact with each other and may develop entanglements as well [22]. In sum, the relationships between c, $c^{*}$, and $c_{e}$ are fundamental to determine whether the polymer solutions would form filaments during ejection.

In this work, we further delve into the physics underlying the fragmentation process due to the micro-mixing and on the size of the resulting ejecta. Recently, we showed that the boundary layer thickness in the interior of the atomizer, in the micro-mixing zone, is the fundamental scale determining the size of the ejecta [23]. The major parameters controlling the process are the gas overpressure (ΔP) and the liquid flow rate (Q). We show that there are at least two ejection types based on a combination of viscosity and liquid flow rate which result in different ejecta’s patterns.

## 2. Materials and Methods

### 2.1. Materials and Preparation of Solutions

Poly(ethylene oxide) (PEO) with a volume-averaged molecular weight ($M_{v}$) of 1,000,000 g/mol (PEO1M) was purchased from Sigma-Aldrich and used as received. To prepare the solutions, an appropriate amount of polymer was dissolved in distilled water (milli-Q) and mixed using a magnetic stirrer, typically for one day and applying mild heating to enhance uniform dissolution. The solutions were cooled at room temperature prior to use. Solutions with concentrations, c, of 0.5 wt.%, 0.8 wt.%, 1.5 wt.%, and 1.6 wt.% were prepared. For atomization experiments a commercial FB atomizer (Ingeniatrics Tecnologías, Seville, Spain) with Ψ=1/7 was used as in our previous studies [23,24,25].

### 2.2. Characterization of Polymer Solutions

The shear viscosity (μ) was measured with a Discovery HR-3 rheometer (TA Instruments) using a cone (angle = 2.009°) and plate (diameter of 60 mm) geometry and a gap of 52 µm. In addition, the instrument is equipped with a Peltier plate to allow control of temperature. All measurements were performed at room temperature and ambient relative humidity (in the range of 50–60%). Solutions were allowed to settle for a sufficiently long time prior to performing the measurements. The zero-shear viscosity ($\mu_{0}$) was taken as the viscosity corresponding to the lowest shear rate. The surface tension (σ) of the solutions was measured with a KSV contact angle meter (CAM 100) configured in a pendant drop geometry, for measurements in the range 0°−180°. The instrument is coupled with a FireWire video camera having a resolution of 640 × 480 pixels. The camera uses telecentric objective lens with a 55 mm focus length. The surface tension is calculated by fitting a curve with the Young-Laplace equation. The solutions’ density, $\rho_{l}$, was measured by weighting a known volume of the liquid.

### 2.3. FB Atomization and Video Processing

**Figure 3:**
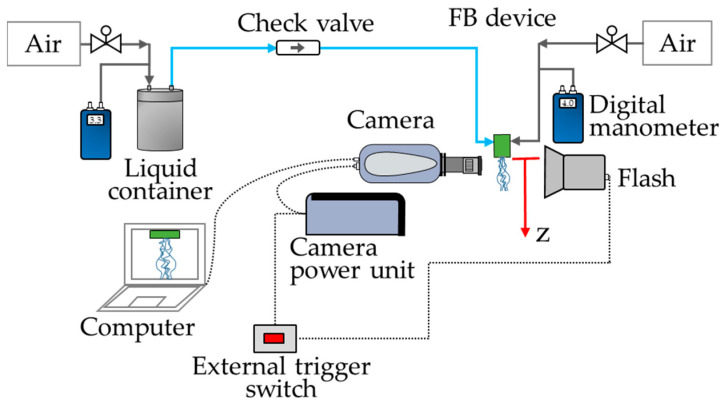
Setup for liquid ejection with the micro-mixing process in a vertical configuration.

The typical setup used for atomization experiments is shown in Figure 3 and is similar to that in our previous publications [23,24,25]. In this case, the atomizer was mounted on an optical table using high-precision components, which allowed its vertical displacement, up and down. The FB atomizer functioned by controlling the overpressures of the lines supplying air (ΔP) and liquid ($\Delta P_{l}$) (as shown in Figure 3), which were measured by digital manometers. The liquid was fed pneumatically through a pressurized liquid container. The FB atomizer was operated by first setting ΔP in the range 3–4 bar, and then adjusting ${\Delta P}_{l}$ to obtain a continuous liquid ejection. The liquid flow rate, *Q*, of each experiment was obtained by weighting a collected volume of polymer solution in a given time period.

The liquid ejection phenomenon was recorded with a Shimadzu HPV-2 video camera (speed of up to 1 million fps) and illuminated with a Walimex Pro Studio Flash (VC-4000) using an opaque glass as diffuser. The videos were recorded at different vertical distances (z) from the exit of the atomization device and the recording plane was fixed along its centerline. The HPV-2 camera, controlled through the manufacturer’s software, began recording 1 ms after an external trigger, connected also to the flash, was manually activated. The videos were processed with the software ImageJ to adjust their brightness/contrast prior to measurement of filament diameters using a machine-learning algorithm developed in-house. The program was trained to detect only well-focused ejecta and allowed to process the hundreds of frames of a video in a few minutes.

## 3. Results and Discussion

### 3.1. Rheology of Solutions

The rheology of polymer solution, which is fundamental in liquid atomization processes, is rather complex and depends on factors such as the type of solvent, temperature, and pressure [26]. The solutions’ properties at concentrations used herein are summarized in Table 1. These concentration values were selected because they are either incipient concentrations for semi-dilute solutions [24,25], just were polymer chains’ interactions become relevant, or concentrations were chains are known to strongly interact.

As shown in Table 1, except for the 0.5 wt.% solution all concentrations used herein are above the coil overlap concentration $c^{*}$, in particular the 1.5- and 1.6 wt.% solutions. $c^{*}$ is the critical concentration above which the polymer coils overlap in solution, it thus represents the fraction of a polymer molecule in a volume of solvent occupied by its coil. This value has been estimated as, $c^{*}=M_{v}/R_{g}^{3}N_{A}$ where $R_{g}=0.0215M_{v}^{0.583}$ (as reported in [27]) is the radius of gyration of the polymer coil (in units of nm) and $N_{A}$ is Avogadro’s number (see [27,28,29] for details). Overlapping of polymer coils is key in filament formation with FB, since if $c/c^{*}<1$, droplets will be ejected from the atomizer as reported by Hermosín-Reyes et al. [25]. Conversely, filaments will be ejected if $c/c^{*}$ is at least of order 1 [23,24]. In the present work, the concentration regime where $c/c^{*}>1$ is analyzed and thus filament generation is expected. Notice that if $c/c^{*}>1$, the solutions’ concentration is considered to be within the so-called semidilute regime [30,31,32,33] where intermolecular interactions and entanglements are relevant.

**Figure 4:**
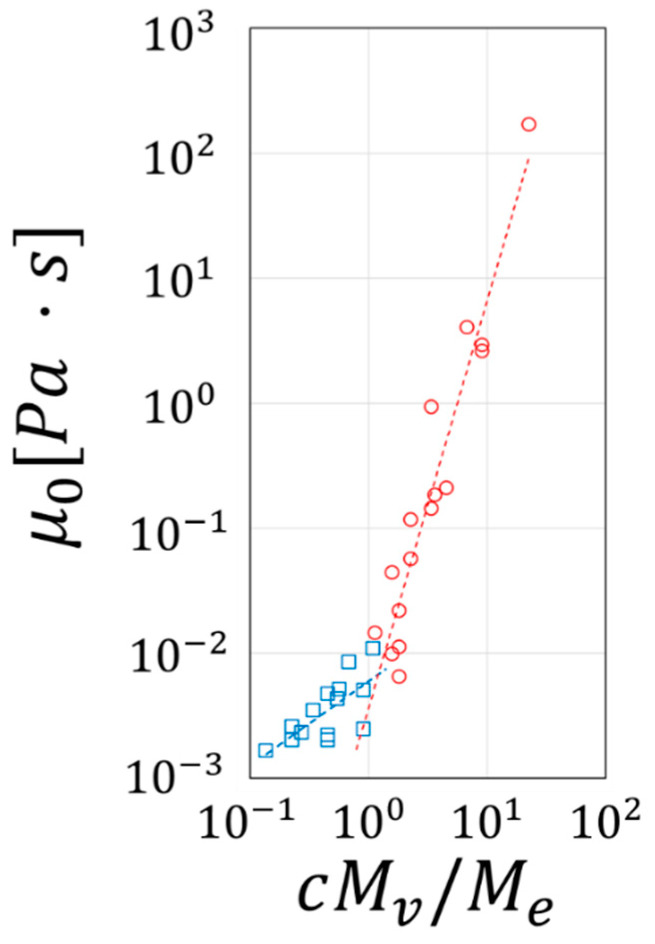
Zero-shear viscosity as a function of the dimensionless parameter $cM_{v}/M_{e}$. Blue squares: dilute regime; red circles: semidilute regime.

Transition from dilute to semidilute solutions is typically represented by a plot of the zero-shear viscosity ($\mu_{0}$) as a function of the polymer molecular weight ($M_{v}$). However, using PEO of varying molecular weights, we have reported $\mu_{0}$ as a function of $cM_{v}/M_{e}$ as shown in Figure 4 and where $M_{e}$ is the critical molecular weight at which polymers form entanglements (4400 g/mol for PEO). The blue squares correspond to data points of the dilute regime while the red circles show those of the semidilute regime. Figure 4 depicts that a clear transition from the dilute regime to the semidilute regime takes place at a value of $cM_{v}/M_{e}$ of order unity. The polymer concentrations used in our experiments give values of $cM_{v}/M_{e}$ > 1.

Another parameter to take into account is the so-called entanglement concentration, $c_{e}$, the critical concentration above which the polymer coils begin to form entanglements, which has been calculated for all solutions with $n_{e}^{3\left(\nu -1\right)}/\left(M_{v}A_{2}\right)$. $n_{e}$ is the number of monomers between entanglements and is obtained as the ratio of the molecular weight of entanglement ($M_{e})$ to the molecular weight of the monomer ($M_{o}$), that is $M_{e}/M_{o}$ [24]. For PEO in water, the exponent is ν=0.583 and $A_{2}=0.0184M_{v}^{-0.2}$ (in mL mol/g^2^) [27]. In this work, the polymer solutions are not expected to form entanglements because $c<c_{e}$, and thus they remain as semidiluted, unentangled solutions [33], as shown in Table 1. Nevertheless, coil overlapping is considered to be a significant factor influencing the dependency of $\mu_{0}$ on $M_{v}$. We later show that these rheologically different behaviors between solutions of relatively low and high concentration give rise to two main ejection patterns with FB.

### 3.2. Filament Ejection and Diameter

**Figure 5:**
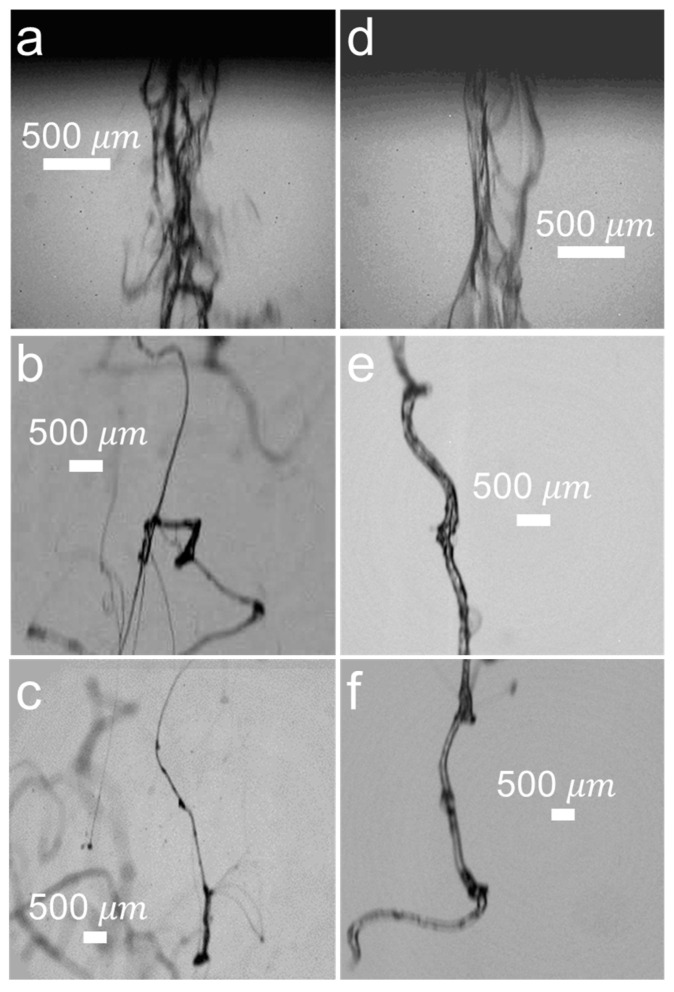
left side: PEO-1M 0.5 wt.% filaments, ΔP=4 bar and Q=0.32 mL/s; right side: PEO-1M 1.5 wt.% filaments, ΔP=4 bar and Q=0.11 mL/s at varying values of z = 0 cm (**a**,**d**), 2 cm (**b**,**e**), and 4 cm (**c**,**f**).

The dynamics of filament ejection was systematically studied as a function of the distance from the atomizer outlet z for two solutions clearly showing distinct rheological behavior. Figure 5 shows images recorded with the HPV-2 camera at z=0 cm, z=2 cm, and z=4 cm, and ΔP=4 bar for Q=0.32 mL/s and Q=0.11 mL/s corresponding to the 0.5 wt.% and 1.5 wt.% solutions, respectively ($\Delta P_{l}∼3.3\;\mathrm{bar}$ in both cases).

The 0.5 wt.% solution’s stream is observed to exit the device partially fragmented and containing voids within the liquid core (Figure 5a), which implies that the FB phenomenon is taking place in the atomizer interior, due to the turbulent mixing between gas and liquid streams. In this case, analyses of videos with the image processing software yield a liquid stream’s mean speed of approximately 300 m/s. Under such exiting conditions it is difficult to discern individual filaments and thus at z=0 cm diameter measurements were not possible. Downstream, in Figure 5b (z=2 cm), some of those threads have clearly separated from the core bundle and simultaneously undergo elongation due to inertial effects, thereby yielding a range of filaments diameters that can be readily measured. Typically, the relaxation length in turbulent cylindrical jets at intermediate Reynolds numbers is known to be of the order of z∼20D [34], which in this work would be z=1.4 cm. Therefore, is thus reasonable to assume that liquid filaments have reached their relaxation length. Even further downstream, as depicted in Figure 5c, the filaments’ separation is enhanced drawn by inertia of the liquid. The filaments have relatively long spanning distances of up to 1 cm.

Conversely, the 1.5 wt.% solution exhibits a strikingly different behavior. As depicted in Figure 5d, the liquid exits as a sole entity and does not seem to experience any fragmentation, resembling a bundle of liquid sheets rather than filaments. However, at z=2 cm as shown in Figure 5e, the liquid sheet has coalesced into a single, relatively thick filament of approximately 300 μm in diameter. It appears as though the inertia of the liquid itself pulls down the sheet and it does not break but rather it oscillates and spins around forming the single filament. Further downstream at z=4 cm (Figure 5f), the filaments appear to maintain its structure and do not experience significant breakup. Occasionally, smaller ejecta are observed at z=4 cm crossing at relatively high speed, thus indicating that they originated further upstream—perhaps they were already fragmented when they exited the device.

**Figure 6:**
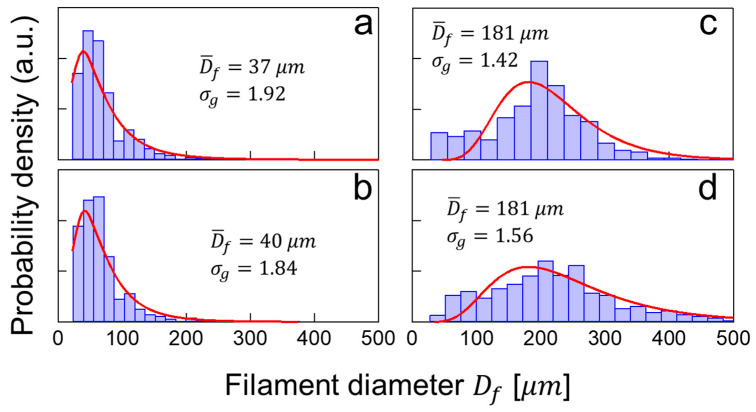
PEO-1M filament size distribution, at z=2 cm, of solutions ejected with ΔP=bar, corresponding to concentrations of (**a**) 0.5 wt.%, (**b**) 0.8 wt.%, (**c**) 1.5 wt.%, and (**d**) 1.6 wt.%.

To quantify the diameter of filaments ejected from liquids of varying viscosities, videos recorded at ΔP=4 bar were analyzed with an in-house developed machine learning algorithm, as performed in our recent publication [23]. Figure 6 shows filament size distributions at z=2 cm for solutions of PEO1M of varying concentrations, where the red line depicts a lognormal distribution fitting. Filaments from the 0.5 wt.% solution show a geometric mean filament diameter of ${\bar{D}}_{f}=37\;\mu m$ and a geometric standard deviation $\sigma_{g}=1.92$ (Figure 6a). Similarly, filaments from the 0.8 wt.% solution show a ${\bar{D}}_{f}=40\;\mu m$ and $\sigma_{g}=1.84$ (Figure 6b). Indeed, these two solutions, which are in the limit of the dilute regime (see Figure 4) with $cM_{w}/M_{e}$ near 1, exhibit similar filament characteristics and dynamics. Their $\sigma_{g}$ are representative of so-called polydisperse distributions which typically have $\sigma_{g}>1.4$, thus indicating the inherently chaotic nature of the breakup process. The polydispersity of filament diameters may be associated with the varying length scales triggered by the turbulent, micro-mixing mechanism [23]. Indeed, such filaments’ diameters are influenced by a combination of the solutions’ flow rate and viscosity with a mayor fraction of them having diameters below 100 μm. Furthermore, solutions with higher polymer concentration were atomized under similar overpressure conditions, resulting in Q=0.11 mL/s and Q=0.085 mL/s, for the 1.5 wt.% and 1.6 wt.% solutions, respectively. In both situations, the large peak below 100 μm observed in low-concentration solutions has shifted toward larger sizes consistent with the observations of Figure 5. The lognormal fitting of the distributions yields a ${\bar{D}}_{f}=181\;\mu m$ with a $\sigma_{g}=1.42$ (Figure 6c) and a ${\bar{D}}_{f}=181\;\mu m$ with a $\sigma_{g}=1.56$ (Figure 6d) for the 1.5 wt.% and 1.6 wt.% solutions, respectively. Notice that the polydispersity of the filaments was significantly reduced, compared to the previous two solutions, as indicated by their $\sigma_{g}$ with an apparent tendency towards monodispersity. It is plausible to think that ejection conditions can lead to variations in the diameter of the filaments.

Recently, based on a scaling analysis of the so-called boundary layer and Kolmogorov’s energy cascade theory, two main liquid ejection types were identified [23]. It is noteworthy to mention that in the current system, values of the so-called Bond number ($Bo=\rho_{l}gD^{2}/\sigma$, i.e., the ratio between gravitational and surface tension forces) are smaller than one, thus indicating negligible gravitational effects for any length scale smaller than D.

**Figure 7:**
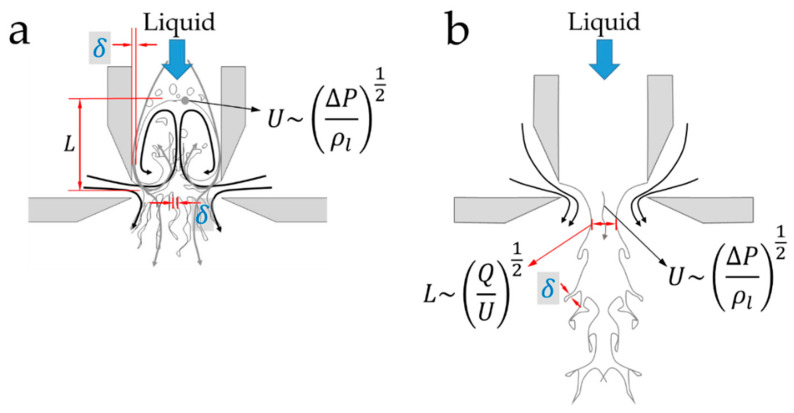
Liquid ejection of (**a**) type I and (**b**) type II. δ is the thickness of the boundary layer and L is a characteristic length scale accordingly defined.

In one type of ejection (type I, Figure 7a), as the gas implodes into the inner tube and forms a cavity, the liquid develops a boundary layer. Its thickness, δ, may be estimated from the classical case of a laminar boundary layer since, given the geometrical constrains, the length scale is relatively short for the flow to develop a turbulent boundary layer. In such case, $\delta ∼L/\sqrt{Re}$; L being a characteristic length scale of the order of the gas cavity, and Re the Reynolds number, defined as in reference [16],

(1) $$Re={\left(\rho_{l}\Delta PD^{2}/\mu^{2}\right)}^{1/2}.$$

Notice that Equation (1) adopts the format of the classical definition of the Reynolds number if one makes use of $U∼{\left(\Delta P/\rho_{l}\right)}^{1/2}$. In this case, U represents a characteristic velocity of the liquid that arises from the overpressure ΔP in the interior of the device. Therefore, U establishes the order of magnitude of the velocity values that the liquid is expected to take in the surroundings of the discharge zone (see Figure 7). Furthermore, here δ may be regarded as the length scale through which an energy rate per unit mass, $U^{3}/\delta$, is incepted into the two-phase flow as described by Kolmogorov’s theory [35]. Based on Kolmogorov’s premise, if we assume that the filament diameter $D_{f}$ is the scale at which the energy rate per unit mass is finally dissipated by velocity fluctuations $u^{*}$, we can then relate

(2) $$U^{3}/\delta ∼u^{*}{}^{3}/D_{f}.$$

Then, the average pressure fluctuations due to $u^{*}$ within the filaments may be balanced with Laplace pressure, such that

(3) $$\rho_{g}u^{*}{}^{2}∼\sigma /D_{f},$$

where $\rho_{g}$ is the gas density. Combining Equations (1)–(3) and solving for $D_{f}$ we obtain that the filaments’ diameter scales as,

(4) $$D_{f}∼\left(\sigma /\rho_{g}U^{2}\right)Re^{-1/5}.$$

Subsequently, dividing $D_{f}$ by the right-hand side of Equation (4) results in a dimensionless filament diameter, as in Equation (5):

(5) $$D_{f}^{*}=D_{f}\;Re^{1/5}/\left(\sigma /\rho_{g}U^{2}\right),$$

thus indicating that $D_{f}^{*}$ should be of order 1. Conversely, in a different type of ejection (type II, Figure 7b) the inertia of the liquid is sufficiently high to prevent the formation of a cavity in the interior of the liquid feeding tube. In that case, the characteristic length scale L is dictated by the liquid flow rate Q and the velocity adopted by the liquid in the vicinity of the discharge region U (Figure 7b), that is,

(6) $$L∼{\left(Q/U\right)}^{1/2},$$

and thus, the thickness of the boundary layer may be estimated as

(7) $$\delta ∼{\left(Q/URe\right)}^{1/2}.$$

Combining Equations (2), (3) and (7) and solving for $D_{f}$ yields,

(8) $$D_{f}∼{\left(\rho_{g}^{2}U^{3}Q/\sigma^{2}\right)}^{1/5}\left(\sigma /\rho_{g}U^{2}\right)Re^{-1/5}.$$

Here, it is convenient to express the liquid flow rate in non-dimensional form, that is,

(9) $$Q^{*}∼\rho_{g}^{2}U^{3}Q/\sigma^{2},$$

and substituting Equations (4) and (9) into (8) yields,

(10) $$D_{f}^{*}∼Q^{*}{}^{1/5}$$

**Figure 8:**
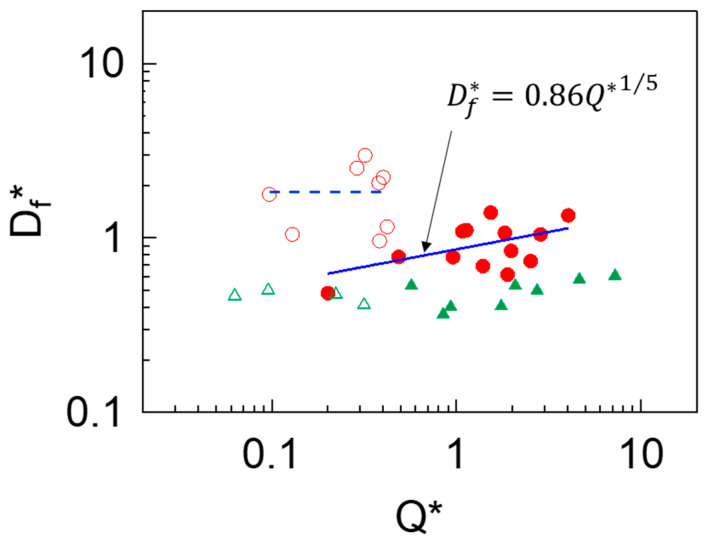
Scaling law of PEO filaments (circles). Data of PVA are included for comparison (triangles).

In Figure 8, we have plotted data of $D_{f}^{*}$ obtained from experiments using PEO of varying molecular weights. The scaling law of $D_{f}^{*}$ (continuous, blue line) represents the experimental data very well (filled, red circles), also agreeing with results from experiments using poly(vinyl alcohol) (PVA) reported by Ramos-Escobar et al. [23], which are also included for comparison (filled, green triangles). In the present case $Q^{*}∼1$ defines the limit between the two types of liquid ejection and filament formation regimes, also agreeing with the data of PVA. It is noteworthy to mention that for solutions with $c<c^{*}$, the values of $\mu_{0}$ reported in the present work are in the same range as those in Ramos-Escobar et al. [23]; however, for cases with $c>c^{*}$ the $\mu_{0}$ of PEO solutions used herein are one order of magnitude higher than those of the PVA experiments. Such difference, in addition to the stearic conformation of polymer chains within the filaments owed to their particular physicochemical properties (e.g., hydrogen bonding, coil structure, etc.) are likely to influence the diameter of the ejecta, which in turn give origin to the slight discrepancies between both polymers observed in Figure 8. In the plot, the only variation between PEO and PVA fittings is the prefactor, which in the case of PVA is ∼0.4 (see Figure 9 in [23]). In a different type of ejection (open circles), $D_{f}^{*}$ values oscillate around constant value of approximately 1.8. This variation may be attributed to fluctuations of the liquid ejecta due to a combination of liquid flow rate and viscosity.

## 4. Concluding Remarks

Unentangled solutions of PEO, in the semidilute regime, where $c∼c^{*}$, were pneumatically ejected using a Flow Blurring device. The atomizer relies on a mixing mechanism, at the micro-meter length scale, taking place in the device interior itself, in the vicinity of the liquid discharge region. We identified two main modes of liquid ejection, whose appearance are dictated by a combination of liquid flow rate and viscosity. In one type, the liquid flow is fragmented in the device interior, in which case the boundary layer thickness is the fundamental length scale determining the size of the ejecta. Conversely, if the inertia of the gas is not sufficiently high to fragment the liquid flow from the device interior, the polymer solution is ejected as a single entity whose characteristic size scales with flow rate. To further understand the physics underlying the ejection phenomena a scaling analysis was performed based on the concepts of the energy cascade proposed by Kolmogorov. The analysis elucidated that the filaments follow a scaling law of the form Df*∼Q*15 where $D_{f}^{*}$ and $Q^{*}$ are dimensionless parameters representing filament diameter and liquid flow rate, respectively. The scaling law fits experimental results very well and thus show that the filaments formed downstream of the atomization device inherently contain the signatures of their ejection settings. Furthermore, the analysis indicates that the parametric frontier between the two types of ejections occurs when $Q^{*}\;$is of order unity. For $Q^{*}$ values smaller than 1, $D_{f}^{*}$ adopts a nearly constant value of approximately 1.8. The approach presented herein aims at elucidating the physics behind the ejection of polymer solutions using FB atomizers, which work with relatively high flow rates and, thus, may be suitable for scaled-up applications.

## Figures and Tables

**Table 1:** Properties of PEO1M aqueous solutions at different concentrations, c.

c(wt%)	$\rho_{l}$ $\left(Kg/m^{3}\right)$	$\mu_{0}$ (Pa⋅s)	σ (N/m)	$\frac{c_{m}M_{v}}{M_{e}}$ $\left(-\right){\;}^{1}$	$c^{*}$ (wt%)	$c_{e}$ (wt%)	${\bar{D}}_{f}$ $\left(µm\right){\;}^{2}$	$\sigma_{g}{\;}^{3}$
0.5	990.9	0.025	0.0582	1.14	0.54	2.71	37	1.92
0.8	993.2	0.043	0.0567	1.82			40	1.84
1.5	999.0	0.900	0.0570	3.41			181	1.42
1.6	994.7	1.050	0.0575	3.64			181	1.56

^1^ $M_{v}$ = 1,000,000 g/mol and $M_{e}$ = 4400 g/mol. ^2^ Geometric mean filament diameter measured at 2 cm from the atomizer outlet and at gas overpressure of 4 bar. ^3^ Geometric standard deviation.

## Data Availability

Data sharing is not applicable to this article.
